# Application of the multiplanar fracture redactor in the treatment of tibial shaft fractures with intramedullary nails

**DOI:** 10.1038/s41598-021-87913-5

**Published:** 2021-04-19

**Authors:** Kuo Zhao, Hongzhi Lv, Chun Zhang, Zhongzheng Wang, Zhiyong Hou, Wei Chen, Qi Zhang, Yingze Zhang

**Affiliations:** 1 Department of Orthopaedic Surgery, Third Hospital of Hebei Medical University, No. 139 Ziqiang Road, Shijiazhuang, Hebei 050051 People’s Republic of China; 2 Key Laboratory of Biomechanics of Hebei Province, Shijiazhuang, 050051 Hebei People’s Republic of China; 3 Orthopaedic Research Institution of Hebei Province, Shijiazhuang, 050051 Hebei People’s Republic of China; 4 NHC Key Laboratory of Intelligent Orthopaedic Equipment (The Third Hospital of Hebei Medical University), Shijiazhuang, People’s Republic of China

**Keywords:** Trauma, Fracture repair

## Abstract

This prospective study aimed to introduce the application of the multiplanar fracture redactor (MFR) in the treatment of tibial shaft fractures with intramedullary nails (IMNs). From February to June 2018, a total of 18 patients with tibial shaft fractures were recruited. MFR was used to help achieve the reduction of tibial shaft fractures with IMN in all patients. The demographic and fracture characteristics, surgical data, postoperative complications and prognostic indicators of 16 patients were recorded. All operations were performed under closed reduction, excellent radiological and functional outcomes were observed. The average duration of surgery, intraoperative blood loss, intraoperative fluoroscopy times, number of intraoperative assistants, and duration of postoperative hospital stay were 91.2 ± 26.1 min, 95.0 ± 58.3 ml, 19.2 ± 2.3 times, 1 (1–2), and 7.8 ± 2.6 days, respectively. The mean Lysholm Knee Function Score (LKFS), American Orthopaedic Foot and Ankle Society (AOFAS) and visual analogue scale (VAS) scores at one year after surgery were 96.8 ± 2.1, 94.8 ± 2.9, and 1 (0–3), respectively. Wound infection, non-union, malunion or complications associated with MFR were not observed in this study. Thus, MFR was a safe and neater method to achieve and maintain the reduction of tibial shaft fractures with IMN.

## Introduction

Tibial shaft fracture is one of the most common fractures of long bone, accounting for 24.75% of tibiofibular fractures and 4% of all adult fractures^1, 2^. It has been reported that 17 per 10,000 persons suffer tibial shaft fractures annually, the rate of which increases sharply with increasing motorization in low- and middle-income countries^3^. The occurrence of tibial shaft fractures exhibits a bimodal age distribution with one peak noted in young patients suffering from high-energy injury and another peak noted in geriatric patients undergoing a low-energy injury^4^. Intramedullary nailing (IMN), plating and external fixation are available options for the surgical treatment of tibial shaft fracture patients^5^. For better biomechanical and biological performances compared with the other alternative fixation methods, IMN has become the preferred method for the surgical treatment of tibial shaft fractures^6–9^. Patients with tibial shaft fractures demonstrated satisfactory functional outcomes and low levels of complications due to their less invasive nature and potential for earlier weight bearing^10, 11^.

Closed reduction and minimally invasive fixation are the ideal goals for the application of IMN in the treatment of tibial shaft fractures. The process and sustained reduction were critical to the surgery. In contrast to femur shaft fractures, for which the traction bed could be used to achieve reduction, tibial shaft fractures were mainly reduced by manual traction^12, 13^. Therefore, open reduction is always required to achieve acceptable reduction in the treatment of tibial shaft fractures with IMN for powerless manual traction, especially for the multi-fragmentary fractures of the tibial shaft^14–17^. Postoperative malalignment is a common complication in tibial shaft fracture patients fixated by IMN, the incidence of which ranges from 19 to 41% as reported by previous studies^18, 19^. As a preventable iatrogenic complication, postoperative malalignment could be decreased or avoided if reduction devices exist to achieve and maintain reduction at the insertion of IMN. To resolve these problems, many methods have been proposed in previous studies^14^. Tubular external fixator as a reduction device has been described but was limited to maintaining the reduction of fractures in only one plane^20^. Blair et al. successfully applied a simple half circular reduction device to achieve the reduction of tibial fractures, but the device was restricted to reduction control in one plane^21^. Anton et al. proposed a new surgical technique of fixator-assisted nailing in the treatment of tibial fracture^22^. The advantages of this technique were that it could control the reduction in the coronal plane and sagittal plane. However, the disadvantages of these methods, including the increased duration of surgery, more difficult surgical techniques and increased medical costs, were not conducive to their popularization. Moreover, most previous studies on reduction devices in the treatment of tibial shaft fractures treated with IMN have primarily concentrated on how to maintain reduction during surgery, and they have limited influence on the reduction of fractures^6, 20, 22^.

To facilitate the closed reduction of tibial shaft fractures and maintain the appreciated alignment on the insertion of the IMN, we designed a multiplanar fracture redactor (MFR) for the treatment of tibial shaft fractures with IMN. The purposes of this study were as follows: (a) to demonstrate the application of MFR in the treatment of tibial shaft fractures with IMN and (b) to assess the outcomes of tibial shaft fractures with IMN facilitated by MFR.

## Patients and methods

**Figure 1:**
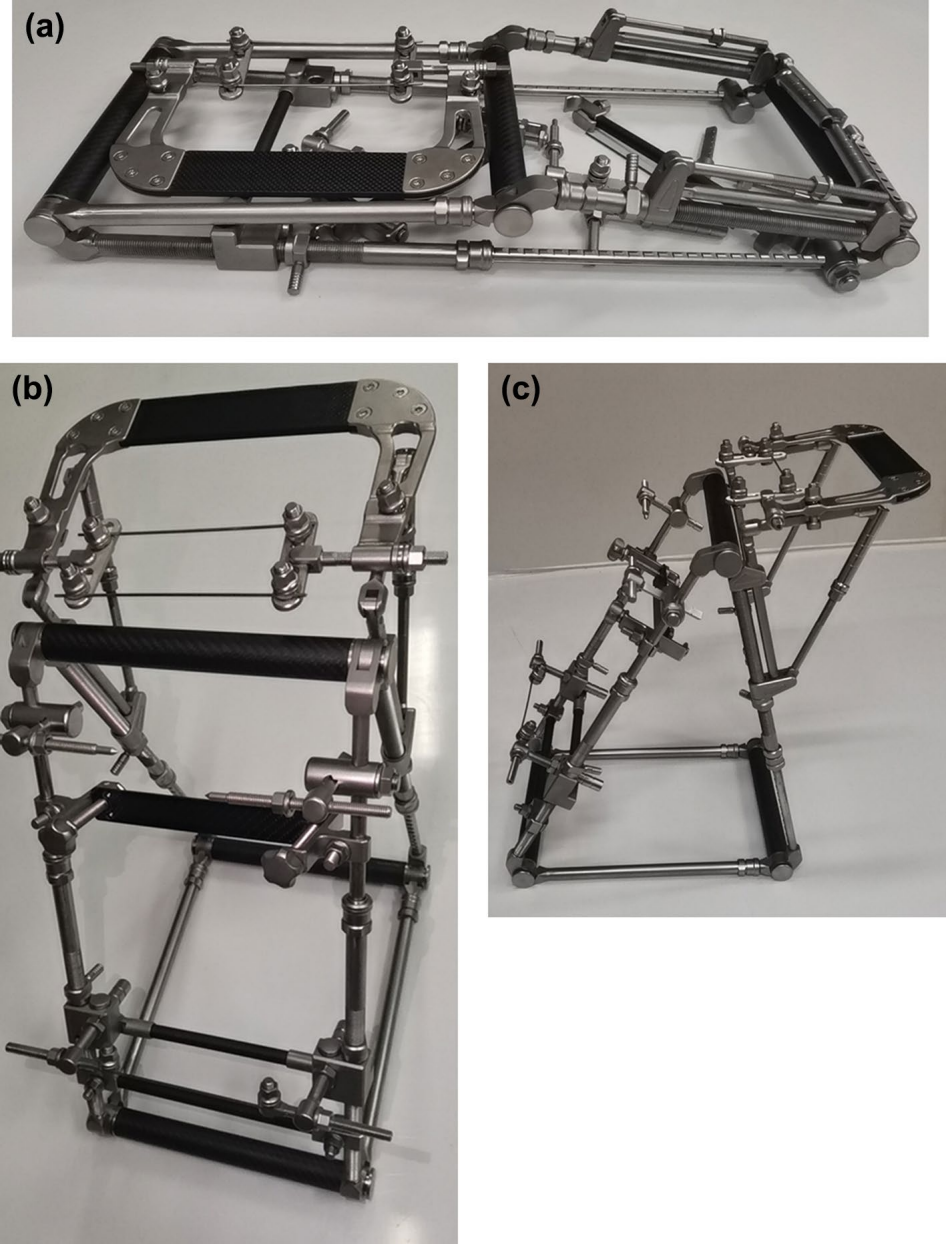
The photos of a multiplanar fracture redactor. (**a**) The view of before installation, (**b**) the antero-posterior view, (**c**) the lateral view.

This prospective study was conducted at a level I trauma centre in a tertiary university hospital. From February to June 2018, patients with tibial shaft fractures who were hospitalized in our department were included in our research. The exclusion criteria were patients with (a) age < 18 years; (b) multiple fractures or open fractures; (c) old fractures (time from injury to surgery > 21 days); and (d) the follow-up < 1 year. All tibial shaft fractures were fixed by IMN with MFR (Fig. 1), and all operations were conducted by the same team, which consisted of 1 chief physician and 2 attending physicians. The ethical committee of Third Hospital of Hebei Medical University approved this study, and all patients in this study signed informed consent before surgery. The ethical standards of the Declaration of Helsinki were followed in the implementation of this study.

### Patients characteristics

A total of 16 continuous patients, including 3 females and 13 males, were recruited in the present study according to the inclusion and exclusion criteria. The patient demographic data and fracture characteristics were shown in Table 1. The mean patient age was 38.9 ± 11.0 years. The AO/OTA classification of the fractures was as follows: 4 cases with type AO/OTA 42 A, 7 cases with type AO/OTA 42 B, and 5 cases with type AO/OTA 42 C. The mean time from injury to surgery was 3.6 ± 1.5 days. Chemoprophylaxis prophylaxis for DVT was adopted at admission for all patients, and routine preoperative examination and preparation were performed prior to surgery. Standard antibiotic prophylaxis in all patients was 1 to 3 g cefazolin sodium through intravenous injection 30 min before surgery.

**Table 1:** Patient demographic data and fracture characteristics.

Variables	Number
Age(years), mean ± SD	38.9 ± 11.0
Gender (male), no. (%)	13(81.2)
Side (left), no. (%)	8(50.0)
**AO classification, no. (%)**	
42 A	4(25.0)
42 B	7(43.8)
42 C	5(31.3)
Injury mechanism (high energy), no. (%)	6(37.5)
Time from injury to surgery(days), mean ± SD	3.6 ± 1.5

*SD* standard deviation.

### Surgical technique

All surgeries were conducted on a radiolucent table, and a supine position was adopted in all patients. The C-arm was located on the contralateral side to facilitate intraoperative fluoroscopy. The tourniquet pressure is uniformly set as 280 mmHg.

#### The introduction of MFR

**Figure 2:**
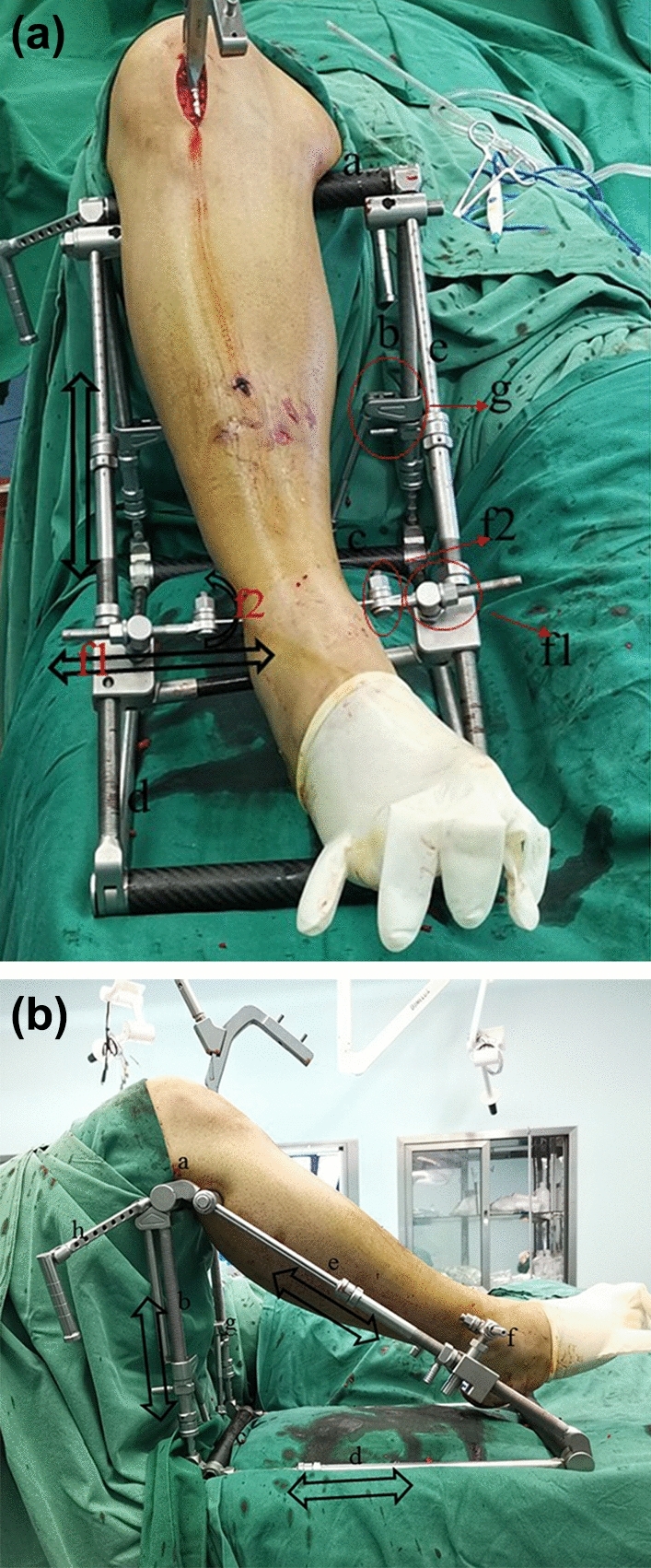
The application of the multiplanar fracture redactor in the treatment of tibial shaft fractures with intramedullary nails, (**a**) The antero-posterior view, (**b**) the lateral view. And the black arrow represents the direction of traction.

The MFR is shown in Fig. 2 and consists of a proximal rod (a), support rod (b), bottom rod (c), traction rod (d), connecting rod (e), distal traction components (f), reset bar (g) and proximal traction components (h). The height of the support rod, the length of the traction rod, the length of the connecting rod, and the position of the distal traction components can be adjusted by intraoperative needs. The reset bar could be used to correct the lateral displacement of the fractures and complete the reduction of the wedge fractures. The distal traction components consisted of the bottom component (f1), middle component (f2) and inside component (f3). The bottom component (f1) controlled the position of the distal traction. The middle component (f2) could achieve lateral adjustment to facilitate lateral displacement. The adjustment of the inside component (f3) would lead to rotation of the K-wire, which would help reduce the rotational deformity. The proximal traction components (h) could be used to provide distal traction in the treatment of femur fracture.

#### Step 1 Reduction

After anaesthesia, skin preparation and draping were conducted. The patients were placed in a supine position with the proximal rod of the MFR under the knee joint. The position of the distal traction components was adjusted according to the length of the calf. The distal traction site was chosen at the calcaneus or above the ankle, and a 2-mm diameter K-wire was chosen as the traction needle. The first step was to restore the length of limb. After installation of the MFR, the MFR was pulled towards the distal end to complete the reset. Most of the shortening displacement could be corrected after traction. For patients with stronger muscles or greater shortening displacement, prolonged, gradual increasing traction could counteract muscle tension, and reduction would be achieved after two or more tractions. After the restoration of limb length, the rotation should be corrected. When the rotation deformity was maintained under fluoroscopy, the inside component (f3) was relaxed, which was conducive to rotating the distal limb to correct the residual displacement. Then, the angular deformity was eliminated by relaxing the middle component (f2) and adjusting the position of the distal limb in the coronal plane.

#### Step 2 Intramedullary nailing

After satisfactory reduction was obtained, the IMN was inserted. First, a 4-cm incision was made above the tuberosity of the tibia. The patellar tendon was cut longitudinally and cleaned the infrapatellar fat pad to expose the tibial plateau slope. The appreciated nail entry point was made, and the guide line was implanted. Different types of reamers were used to expand the medulla in turn. After full expansion of the medulla, a suitable main nail was applied to fix the fractures. The reduction of fracture, the position of the guide line, the size of the main nail, and the fixation of the IMN were evaluated from the C-arm perspective. After inserting the distal locking nail and the tail cap, the MFR was removed. Finally, the position of all the screws was evaluated under the C-arm perspective, and the incisions were sutured.

### Postoperative management

Postoperative radiological evaluation of the tibia was conducted on the day of the surgery. Standard antibiotic prophylaxis in all patients was applied for 24 h to prevent infection. Low molecular weight heparin sodium was injected to prevent the development of deep venous thrombosis on the first day after surgery. Rehabilitation exercises needed to start as soon as possible. Partial weight bearing was permitted six weeks after surgery.

### Data collection

The duration of surgery, intraoperative blood loss, intraoperative fluoroscopy times; number of intraoperative assistants; duration of postoperative hospital stay; in-hospital complications, including postoperative deep venous thrombosis and wound infection; non-union; and malunion were collected. Follow-up was conducted at 1, 3, 6, 9 and 12 months postoperatively, and the radiological and functional assessments were collected at the last follow-up. If a difficulty of fracture healing was observed at 9 months postoperatively and had limited progression in the following 3 consecutive months, it was defined as non-union^23^. Malunion was defined as varus or valgus angulation > 5°, shortening or parallel displacement > 5 mm or rotation deformity > 10°^24^. The radiological evaluation was accomplished through X-ray by one radiologist in our hospital. The functional evaluation, including the Lysholm Knee Function Score (LKFS), visual analogue scale (VAS) and American Orthopaedic Foot and Ankle Society (AOFAS) scores, was completed by one orthopaedic surgeon in our department^25–27^.

## Results

### Surgical comparison

**Figure 3:**
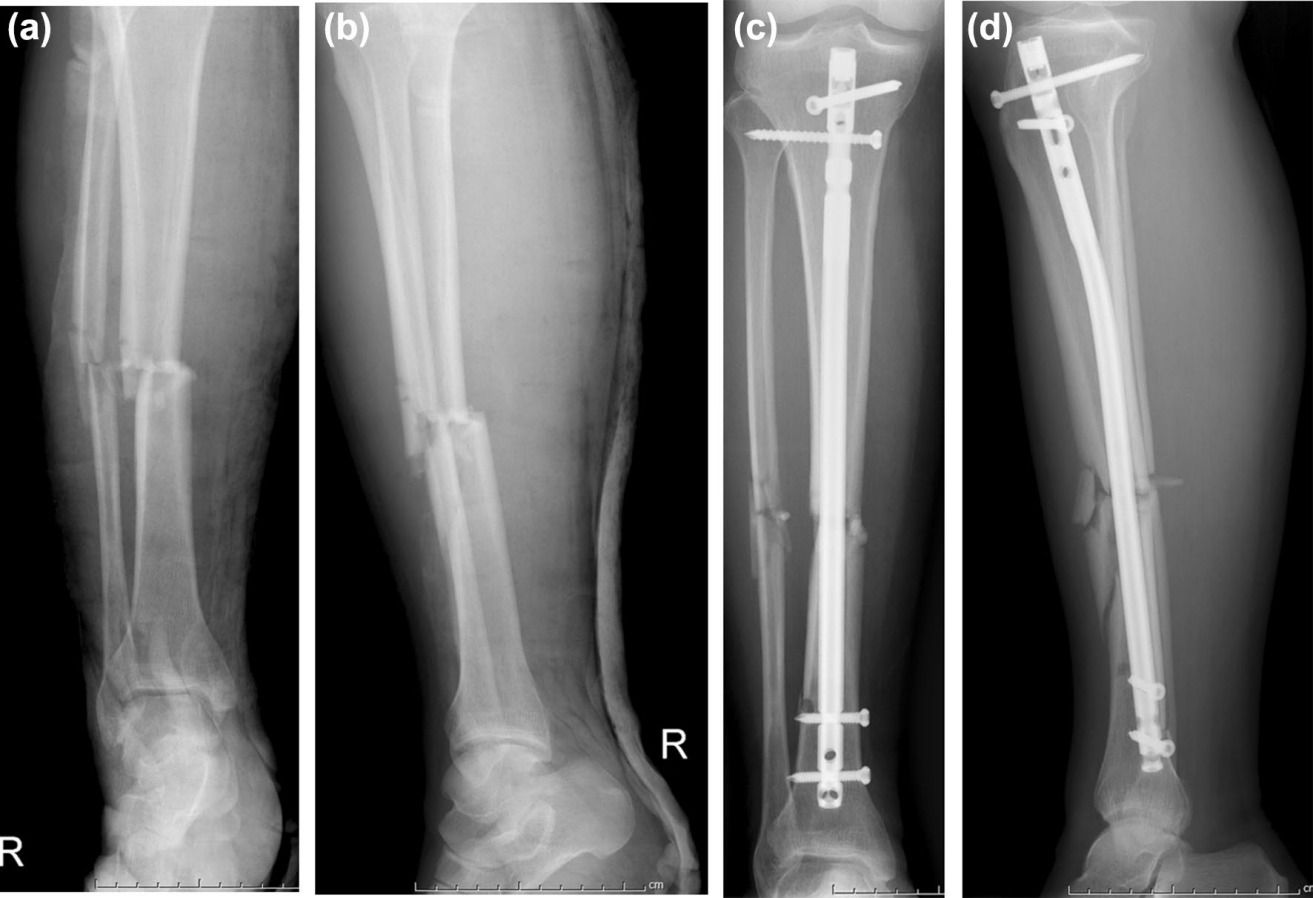
A 38-year-old male with the tibial shaft fracture. (**a**) The antero-posterior X-ray before surgery (**b**) the lateral X-ray before surgery, (**c**) the antero-posterior X-ray after surgery, (d) the lateral X-ray after surgery.

MFR successfully achieved and maintained the reduction of all tibial shaft fractures in this study (Fig. 3). All operations were performed under closed reduction, and excellent alignment was discovered in the postoperative radiological evaluation. Nine surgery were performed under general anesthesia. In this study, 16 patients were followed up for 1 year, and 2 patients were lost to follow-up. The average duration of surgery, intraoperative blood loss, intraoperative fluoroscopy times, number of intraoperative assistants, and duration of postoperative hospital stay were 91.2 ± 26.1 min, 95.0 ± 58.3 ml, 19.2 ± 2.3 times, 1 (1–2), and 7.8 ± 2.6 days, respectively (Table 2). None surgery was conducted by open reduction. Postoperative anteroposterior and lateral X-rays of the tibia demonstrated satisfactory fracture reduction and fixation in all patients.

**Table 2:** Details of surgical data.

Variables	Number
Duration of surgery (min), mean ± SD	91.2 ± 26.1
Intraoperative blood loss (ml), mean ± SD	95.0 ± 58.3
Intraoperative fluoroscopy times, mean ± SD	19.2 ± 2.3
Anesthesia (general), no. (%)	9 (56.3)
Open reduction, no. (%)	0 (0.0)
Number of intraoperative assistants, mean (range)	1 (1–2)

*SD* standard deviation.

### Prognostic comparison

The mean postoperative hospital stays were 7.8 ± 2.6 days in this study (Table 3). The mean LKFS, AOFAS scores and VAS scores at one year after surgery were 96.8 ± 2.1, 94.8 ± 2.9, and 1 (0–3), respectively. Four patients reported pain around the knee. One patient was found to have postoperative deep venous thrombosis before discharge, which was partially recanalized after treatment with low molecular weight heparin. No wound infection was discovered. The fracture healing was observed in all patients, the average time which was 4.1 (range from 3 to 6) months. All patients were observed excellent alignment at the last follow-up, and malunion were not observed in this study. No complications associated with MFR, such as reductor-induced or K-wire-induced neurovascular injury, were discovered in this study.

**Table 3:** Prognostic comparison.

Variables	Number
Postoperative hospital stays, mean ± SD	7.8 ± 2.6
LKFS, mean ± SD	96.8 ± 2.1
AOFAS scores, mean ± SD	94.8 ± 2.9
VAS scores, mean(range)	1(0–3)
Deep venous thrombosis, no. (%)	1(6.3)
Fracture healing time(months), mean(range)	4.1(3–6)

*SD* standard deviation.

## Discussion

Currently, IMNs have become the preferred implants for the surgical treatment of tibial shaft fractures^8, 28, 29^. Compared to other alternative implants, the IMN was associated with some advantages, including early stable fixation for earlier weight bearing and less soft-tissue dissection around the fracture. In contrast to plate fixation, in which accurate alignment would be achieved under direct visualization, malalignment was not rare in the application of IMN^19^. MFR represents a safe method to achieve and maintain the reduction of all tibial shaft fractures in this study.

Excellent alignment was discovered in the postoperative radiological evaluation in all patients in the present study, and all operations were performed under closed reduction in the present study.

Malalignment is a common complication for patients with tibial shaft fractures treated by IMN, which has been reported to account for 19% to 41% of cases^18, 30, 31^. Patients with postoperative malalignment are likely to have a compromised functional outcome^32^. Therefore, postoperative alignment is a critical evaluation index for surgery. Excellent alignment was discovered in the postoperative radiological evaluation in all patients in the present study. The MFR helped achieve good reduction and also maintained the reduction in the insertion of IMN in this study. Compared to manual traction, the traction force produced by the proximal rod and the K-wire in the inferior tibiofibular join was sufficiently powerful to correct the shortening displacement. One of the characteristics of the MFR is that the traction force is skeletal traction, which is powerful and sustainable. For some difficult displacements, the traction force could be increased gradually, and traction could be reduced two or more times. Another advantage of the MFR was that it could correct the lateral and rotation deformities with the help of its traction components (distal traction components (f) and reset bar (g)), which could help complete the reduction in the coronal plane and sagittal plane. In addition, the reduction could be maintained on the insertion of the IMN with the help of the MFR. Therefore, excellent alignment could be achieved in patients in this study.

Closed reduction was a significant advantage in the treatment of tibial shaft fractures with IMN compared to that with plates. However, some types of fractures are difficult to reduce by closed reduction, and closed reduction always increases the operation time, radiation exposure, or suboptimal fracture reduction^33^. Open reduction is a common method for the reduction of tibial shaft fracture, which is always associated with an increased risk of wound infections and nonunion for the destruction of a haematoma and early inflammatory response^34^. To solve the aseptic or infected non-union, many methods were explored^35, 36^. Auston et al. reported a 17.8% open reduction rate in patients with tibial shaft fractures with IMN in their study, and their results demonstrated that the rates of wound complications (3.6%), infection (7.1%), and non-union (7.1%) in the open reduction group were greater than those in the closed reduction group^33^. The high rate of postoperative complications might be related to the stripping of soft tissue and devascularization of fracture fragments^37^. All operations were performed under closed reduction in the present study, which might be attributed to the powerful traction force produced by skeletal traction with the MFR and its traction components. The average number of intraoperative fluoroscopies was 19.2 ± 2.3, and we insisted that MFR could significantly reduce radiation exposure for orthopaedic surgeons. Moreover, the application of MFR could decrease the number of assistants in the surgery, which was associated not only with a lower risk of wound infections but also with lower medical costs^38, 39^. What’s more, the installation and application of the MFR was so simple that it did not require a long learning curve. And the MFR was a neater form of set-ups used in some units when an Ilizarov frame was being applied. Last, the storage and disinfection of it were easy after folding which was convenient for reuse.

There were several limitations in this study. First, the sample size was too small to enrol sufficient patents with tibial shaft fractures, which was difficult to reduce. Second, although excellent results were observed in all patients in this study, a control group with the help of manual traction for reduction was not included. To resolve these problems, a large sample study to compare the efficacy of MFR and manual traction in the treatment of tibial shaft fractures with IMN will be conducted in the future.

## Conclusion

MFR is a safe method to achieve and maintain the reduction of tibial shaft fractures with IMN. This study provided a new choice for orthopaedic surgeons in the minimally invasive treatment of tibial shaft fractures with IMN.

## References

[1] Zhang, Y. *Clinical Epidemiology of Orthopaedic Trauma*. (Thieme, 2016).

[2] Johal H, Bhandari M, Tornetta P (2016). Cochrane in CORR ®: intramedullary nailing for tibial shaft fractures in adults (Review). Clin. Orthop. Relat. Res..

[3] Johal H, Schemitsch EH, Bhandari M (2014). Why a decade of road traffic safety?. J. Orthop. Trauma.

[4] Thompson, J. H., Koutsogiannis, P. & Jahangir, A. In *StatPearls* (StatPearls Publishing Copyright © 2020, StatPearls Publishing LLC., 2020).

[5] Labronici PJ, Santos Pires RE, Franco JS, Alvachian Fernandes HJ, Dos Reis FB (2011). Recommendations for avoiding knee pain after intramedullary nailing of tibial shaft fractures. Patient Saf. Surg..

[6] Lu Y (2020). Tibial shaft fractures treated with intramedullary nailing and reduction device assistance. Int. Orthop..

[7] Busse JW, Morton E, Lacchetti C, Guyatt GH, Bhandari M (2008). Current management of tibial shaft fractures: A survey of 450 Canadian orthopedic trauma surgeons. Acta Orthop..

[8] Stavrou PZ (2016). Prevalence and risk factors for re-interventions following reamed intramedullary tibia nailing. Injury.

[9] Falzarano G (2018). Foot loading and gait analysis evaluation of nonarticular tibial pilon fracture: A comparison of three surgical techniques. J. Foot Ankle Surg..

[10] Connelly CL (2014). Outcome at 12 to 22 years of 1502 tibial shaft fractures. Bone Joint J.

[11] Vallier HA, Cureton BA, Patterson BM (2012). Factors influencing functional outcomes after distal tibia shaft fractures. J. Orthop. Trauma.

[12] Zhang R (2018). Traction table versus double reverse traction repositor in the treatment of femoral shaft fractures. Sci. Rep..

[13] Ricci WM, Gallagher B, Haidukewych GJ (2009). Intramedullary nailing of femoral shaft fractures: Current concepts. J. Am. Acad. Orthop. Surg..

[14] Hak DJ (2011). Intramedullary nailing of proximal third tibial fractures: techniques to improve reduction. Orthopedics.

[15] Rollo G (2019). Challenges in the management of floating knee injuries: Results of treatment and outcomes of 224 consecutive cases in 10 years. Injury.

[16] Meccariello L (2021). A new prognostic pelvic injury outcome score. Med. Glas..

[17] Fortina M (2019). Jockey injuries during the Siena "Palio". A 72-year analysis of the oldest horse race in Italy. Injury.

[18] Theriault B, Turgeon AF, Pelet S (2012). Functional impact of tibial malrotation following intramedullary nailing of tibial shaft fractures. J. Bone Joint Surg. Am..

[19] Minhas SV, Ho BS, Switaj PJ, Ochenjele G, Kadakia AR (2015). A comparison of 30-day complications following plate fixation versus intramedullary nailing of closed extra-articular tibia fractures. Injury.

[20] Belangero WD, Santos Pires RE, Livani B, Rossi FL, de Andrade ALL (2018). “Clothesline technique” for proximal tibial shaft fracture fixation using conventional intramedullary nail: A simple, useful, and inexpensive technique to prevent fracture malalignment. Eur. J. Orthop. Surg. Traumatol..

[21] Blair S, Gregori A (2001). Tibial nailing using an elementary Ilizarov frame. Ann. R. Coll. Surg. Engl..

[22] Semenistyy AA, Litvina Ea EA, Fedotova AG, Gwam C, Mironov AN (2019). Fixator-assisted nailing of tibial fractures: New surgical technique and presentation of first 30 cases. Injury.

[23] Bell A, Templeman D, Weinlein JC (2016). Nonunion of the Femur and Tibia: An update. Orthop. Clin. North. Am..

[24] Wilczek JL, LaPorta GA (2018). The evolution of limb deformity: What has changed over the past ten years?. Clin. Podiatr. Med. Surg..

[25] Schneider W, Jurenitsch S (2016). Normative data for the American Orthopedic Foot and Ankle Society ankle-hindfoot, midfoot, hallux and lesser toes clinical rating system. Int. Orthop..

[26] Lysholm J, Gillquist J (1982). Evaluation of knee ligament surgery results with special emphasis on use of a scoring scale. Am. J. Sports Med..

[27] Jensen MP, Karoly P, Braver S (1986). The measurement of clinical pain intensity: A comparison of six methods. Pain.

[28] Schemitsch EH (2012). Prognostic factors for predicting outcomes after intramedullary nailing of the tibia. J. Bone Joint. Surg. Am..

[29] Bhandari M, Guyatt GH, Tong D, Adili A, Shaughnessy SG (2000). Reamed versus nonreamed intramedullary nailing of lower extremity long bone fractures: A systematic overview and meta-analysis. J. Orthop. Trauma.

[30] Jafarinejad AE, Bakhshi H, Haghnegahdar M, Ghomeishi N (2012). Malrotation following reamed intramedullary nailing of closed tibial fractures. Indian J. Orthop..

[31] Say F, Bülbül M (2014). Findings related to rotational malalignment in tibial fractures treated with reamed intramedullary nailing. Arch. Orthop. Trauma Surg..

[32] Nork SE (2005). Intramedullary nailing of distal metaphyseal tibial fractures. J. Bone Joint Surg. Am..

[33] Auston DA, Meiss J, Serrano R (2017). Percutaneous or open reduction of closed tibial shaft fractures during intramedullary nailing does not increase wound complications, infection or nonunion rates. J. Orthop. Trauma..

[34] Bone LB, Johnson KD (1986). Treatment of tibial fractures by reaming and intramedullary nailing. J. Bone Joint Surg. Am..

[35] Rollo G (2020). Platet Rich Plasma or Hyperbaric Oxygen Therapy as callus accellerator in aseptic tibial non union Evaluate of outcomes. Acta Biomed..

[36] Rollo G (2021). Effectiveness of teriparatide combined with the Ilizarov technique in septic tibial non-union. Med. Glas..

[37] Oni OO, Dunning J, Mobbs RJ, Gregg PJ (1991). Clinical factors and the size of the external callus in tibial shaft fractures. Clin. Orthop. Relat. Res..

[38] Burgers PT (2016). Total medical costs of treating femoral neck fracture patients with hemi- or total hip arthroplasty: A cost analysis of a multicenter prospective study. Osteoporos. Int..

[39] Howard JL, Hanssen AD (2007). Principles of a clean operating room environment. J. Arthroplasty.

